# Succinic acid inhibits the activity of cytochrome P450 (CYP450) enzymes

**DOI:** 10.1080/13880209.2020.1839110

**Published:** 2020-12-17

**Authors:** Hao Wang, Bingyan Xia, Mei Lin, Yongpeng Wang, Bin Sun, Yuzhu Li

**Affiliations:** a Department of Pharmacy, Yantai Affiliated Hospital of Binzhou Medical University, Yantai, China; b Department of Laboratory, Yantai Affiliated Hospital of Binzhou Medical University, Yantai, China; c The outpatient department, Yantai Affiliated Hospital of Binzhou Medical University, Yantai, China; d Department of Cardiovascular Medicine, Yidu Central Hospital of Weifang, Weifang, China; e Department of Emergency, Yidu Central Hospital of Weifang, Weifang, China; f Department of Critical Care Medicine, Yantai Affiliated Hospital of Binzhou Medical College, Yantai, China

**Keywords:** CYP3A4, CYP2D6, CYP2C9, drug-drug interaction

## Abstract

**Context:**

Succinic acid, extracted from amber, is widely used in cardiovascular therapy.

**Objective:**

The effect of succinic acid on the activity of cytochrome P450 (CYP450) enzymes was investigated in this study.

**Materials and methods:**

The effect of succinic acid (100 μM) on the activity of eight isoforms of CYP450 (i.e., 1A2, 3A4, 2A6, 2E1, 2D6, 2C9, 2C19 and 2C8) was investigated compared to the specific inhibitor and blank controls in pooled human liver microsomes *in vitro*. The inhibition of CYPs was fitted with competitive or non-competitive inhibition models and corresponding parameters were also obtained.

**Results:**

Succinic acid exerted inhibitory effect on the activity of CYP3A4, 2D6, and 2C9 with the IC_50_ values of 12.82, 14.53, and 19.60 μM, respectively. Succinic acid inhibited the activity of CYP3A4 in a non-competitive manner with the *Ki* value of 6.18 μM, and inhibited CYP2D6 and 2C9 competitively with *Ki* values of 7.40 and 9.48 μM, respectively. Furthermore, the inhibition of CYP3A4 was found to be time-dependent with the *KI/K_inact_* value of 6.52/0.051 min^−1^·μM^−1^.

**Discussion and conclusions:**

Succinic acid showed *in vitro* inhibitory effects on the activity of CYP3A4, 2D6, and 2C9, which indicated the potential drug-drug interactions. Succinic acid should be carefully co-administrated with the drugs metabolized by CYP3A4, 2D6, and 2C9.

## Introduction

Cardiovascular disease is a ubiquitous heart disease that threatens human health (Gać et al. 2017). With the development of various therapies, traditional Chinese medicine (TCM) has been widely used in the treatment of cardiovascular disease. In TCM, it is common to co-administrated different kinds of drugs in one prescription. Succinic acid is a main component of amber, which is commonly used for the therapy for arrhythmia. Succinic acid has been reported to possess a variety of pharmacological effects, including cardioprotective, antithrombotic, anti-inflammatory, and antibacterial, which make it easier to co-administrated with other drugs (Tang et al. 2013; Zhang et al. 2014; Radkowski et al. 2018; Nissen et al. 2019).

Cytochrome P450 (CYP450) enzymes are responsible for the metabolism of a wide array of endogenous compounds and xenobiotics, which exist mostly in the liver (Uno et al. 2012). Approximately 70–80% of the known Phase I and II metabolism is attributed to CYP450s (Foo et al. 2015; Li et al. 2015). The induction or inhibition of CYP450s might affect the concentration of co-administered drugs in the blood or therapeutic targets and result in adverse effects, which results in the toxicity of drugs or failure of treatment (Kiser et al. 2013). Recently, the *in vitro* effect of various bioactive compounds on the activity of CYP450s has been studied in human liver microsomes, which is closed to the *in vivo* system. However, the specific effects of succinic acid on the activity of CYP450s was not clear, which is necessary for the clinical administration and co-administration of succinic acid.

The activity of CYP1, CYP2, and CYP3 is closely related to the metabolism of exogenous compounds and could be strongly affected by the presence of environmental pollutants and dietary bioactive compounds (Xu et al. 2018). The *in vitro* effect of succinic acid on the activity of CYP1A2, 3A4, 2A6, 2E1, 2D6, 2C9, 2C19, and 2C8 was investigated in pooled human liver microsomes, with the help of their specific substrates: phenacetin (CYP1A2), testosterone (CYP3A4), coumarin (CYP2A6), chlorzoxazone (CYP2E1), dextromethorphan (CYP2D6), diclofenac (CYP2C9), *S*-mephenytoin (CYP2C19) and paclitaxel (CYP2C8). The effect model was also investigated by kinetic studies.

## Materials and methods

### Chemicals

**Figure 1.**
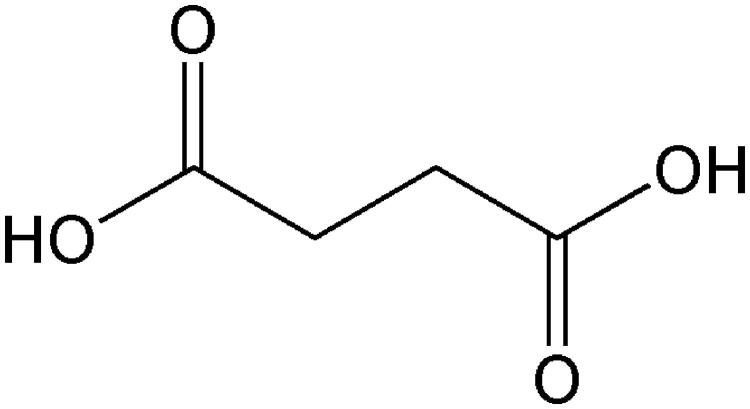
The chemical structure of succinic acid.

Succinic acid (≥98%) and testosterone (≥98%) were obtained from the National Institute for the Control of Pharmaceutical and Biological Products (Beijing, China). The chemical structure of succinic acid is shown in Figure 1. d-Glucose-6-phosphate, glucose-6-phosphate dehydrogenase, corticosterone (≥98%), NADP+, phenacetin (≥98%), acetaminophen (≥98%), 4-hydroxymephenytoin (≥98%), 7-hydroxycoumarin (≥98%), 4′-hydroxydiclofenac (≥98%), sulfaphenazole (≥98%), quinidine (≥98%), tranylcypromine (≥98%), chlorzoxazone (≥98%), 6-hydroxychlorzoxazone (≥98%), paclitaxel (≥98%), 6β-hydroxytestosterone (≥98%), clomethiazole (≥98%), and furafylline (≥98%) were obtained from Sigma Chemical Co (MO, USA). Montelukast (≥98%) was obtained from Beijing Aleznova Pharmaceutical (Beijing, China). Coumarin (≥98%), diclofenac (≥98%), dextromethorphan (≥98%), and ketoconazole (≥98%) were purchased from ICN Biomedicals (Costa Mesa, California). Pooled HLMs were purchased from BD Biosciences Discovery Labware. All other reagents and solvents were of analytical reagent grade.

### Assay with human liver microsomes

As shown in Table 1, to investigate the effects of succinic acid on different CYP isoforms in HLM, the following probe reactions were used, according to the previously described method (Zhang et al. 2007; Qi et al. 2013): phenacetin *O*-deethylation for CYP1A2, testosterone 6β-hydroxylation for CYP3A4, coumarin 7-hydroxylation for CYP2A6, chlorzoxazone 6-hydroxylation for CYP2E1, dextromethorphan *O*-demethylation for CYP2D6, diclofenac 4′-hydroxylation for CYP2C9, *S*-mephenytoin 4-hydroxylation for CYP2C19, and paclitaxel 6α-hydroxylation for CYP2C8. All incubations were performed in triplicate, and the mean values were utilized. The typical incubation systems contained 100 mM potassium phosphate buffer (pH 7.4), NADPH generating system (1 mM NADP^+^, 10 mM glucose-6-phosphate, 1 U/mL of glucose-6-phosphate dehydrogenase, and 4 mM MgCl_2_), the appropriate concentration of HLMs, a corresponding probe substrate and hispidulin (or positive inhibitor for different probe reactions) in a final volume of 200 μL.

**Table 1.** Isoforms tested, marker reactions, incubation conditions, and K_m_ used in the inhibition study.

CYPs	Marker reactions	Substrate concentration (μM)	Protein concentration (mg/mL)	Incubation time (min)	Estimated K_m_ (μM)
1A2	phenacetin *O*-deethylation	40	0.2	30	48
3A4	testosterone 6β-hydroxylation	50	0.5	10	53
2A6	coumarin 7-hydroxylation	1.0	0.1	10	1.5
2E1	chlorzoxazone 6-hydroxylation	120	0.4	30	126
2D6	dextromethorphan *O*-demethylation	25	0.25	20	4.8
2C9	diclofenac 4′-hydroxylation	10	0.3	10	13
2C19	*S*-Mephenytoin 4-hydroxylation	100	0.2	40	105
2C8	paclitaxel 6α-hydroxylation	10	0.5	30	16

The concentration of succinic acid was 100 μM, and the positive inhibitor concentrations were as follows: 10 μM furafylline for CYP1A2, 1 μM ketoconazole for CYP3A4, 10 μM tranylcypromine for CYP2A6, 50 μM clomethiazole for CYP2E1, 10 μM quinidine for CYP2D6, 10 μM sulfaphenazole for CYP2C9, 50 μM tranylcypromine for CYP2C19, 5 μM montelukast for CYP2C8. Probe substrates, positive inhibitors (except for dextromethorphan and quinidine, which were dissolved in water), and succinic acid were dissolved in methanol, with a final concentration of 1% (v/v), and 1% neat methanol was added to the incubations without inhibitor. The final microsomal protein concentration and incubation times for the different probe reactions are shown in Table 1. There was a 3 min preincubation period (at 37 °C) before the reaction was initiated by adding an NADPH-generating system. The reaction was terminated by adding a 100 μL acetonitrile (10% trichloroacetic acid for CYP2A6) internal standard mix, and the solution was placed on ice. The mixture was centrifuged at 12,000 rpm for 10 min, and an aliquot (50 μL) of supernatant was transferred for HPLC analysis. The instrument used in this study was Agilent 1260 series instrument with DAD and FLD detector, and the quantitative assay for the corresponding metabolites was performed as previously reported (Lang et al. 2017; Zhang et al. 2017).

### Enzyme inhibition and kinetic studies of succinic acid

Succinic acid (100 μM) was used to initially screen for its direct inhibitory effects towards different human CYP isoforms. For the CYP isoforms whose activities were strongly inhibited, secondary studies were performed to obtain the half inhibition concentration (IC_50_). *K_i_* values were obtained by incubating various concentrations of different probe substrates (20–100 μM testosterone, 10–50 μM dextromethorphan, 5–20 μM diclofenac) in the presence of 0–50 μM succinic acid.

### Time-dependent inhibition study of succinic acid

To determine whether succinic acid could inhibit the activity of CYP3A4, 2D6, and 2C9 in a time-dependent manner, succinic acid (20 μM) was pre-incubated with HLMs (1 mg/mL) in the presence of an NADPH-generating system for 30 min at 37 °C. After incubation, an aliquot (20 μL) was transferred to another incubation tube (final volume 200 μL) containing an NADPH-generating system and probe substrates whose final concentrations were approximate to *K_m_*. Then, further incubations were performed to measure the residual activity. After being incubated for 0, 5, 10, 15, and 30 min, the reactions were terminated by adding a 100 μL acetonitrile internal standard mix and then placed on ice; the corresponding metabolites were determined by HPLC.

To determine the *KI* and *K_inact_* values for the inactivation of CYP3A4, the incubations were conducted using higher probe substrate concentrations (approximately 4-fold *K_m_* values) and various concentrations of succinic acid (0–50 μM) after different preincubation times (0–30 min), with a two-step incubation scheme, as described above.

### Statistical analysis

The enzyme kinetic parameters for the probe reaction were estimated from the best fit line, using least-squares linear regression of the inverse substrate concentration versus the inverse velocity (Lineweaver-Burk plots), and the mean values were used to calculate *V_max_* and *K_m_*. Inhibition data from the experiments that were conducted using multiple compound concentrations were represented by Dixon plots, and inhibition constant (*K_i_*) values were calculated using non-linear regression according to the following equation:

$$v=(V_{\max}S)/(K_{m}(1+I/K_{i})+S),$$

where I is the concentration of the compound, *K_i_* is the inhibition constant, S is the concentration of the substrate, and *K_m_* is the substrate concentration at half the maximum velocity (*V_max_*) of the reaction. The mechanism of the inhibition was inspected using the Lineweaver-Burk plots and the enzyme inhibition models. The data comparison was performed using Student’s *t*-test and performed using IBM SPSS statistics 20 (SPSS Inc.).

## Results

### Succinic acid inhibits the activity of CYP3A4, 2D6, and 2C9

**Figure 2.**
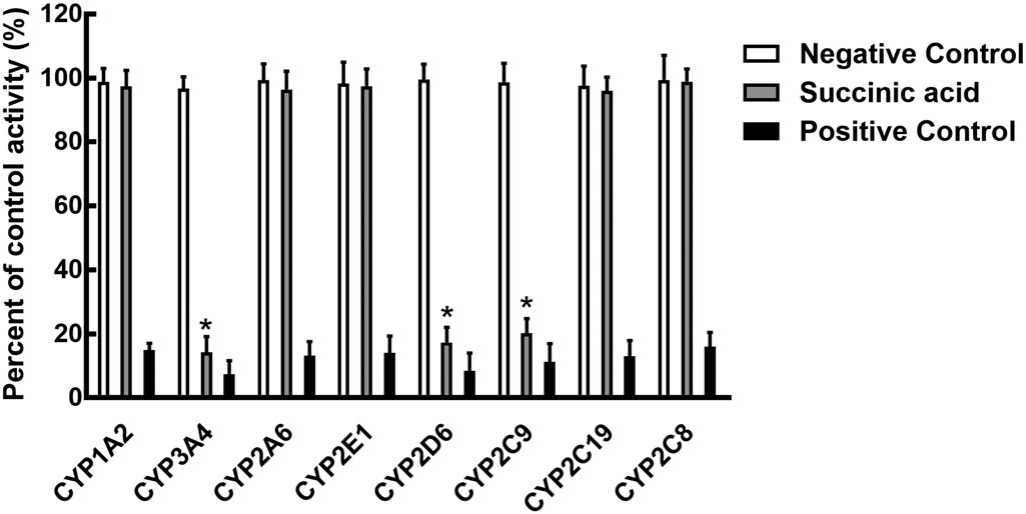
Effects of succinic acid on the activity of CYP450, including CYP1A2, 3A4, 2A6, 2E1, 2D6, 2C9, 2C19, and 2C8. Negative control: incubation without succinic acid. Succinic acid: incubation with 100 μM succinic acid. Positive control: incubation with specific inhibitors. *p < 0.05.

**Figure 3.**
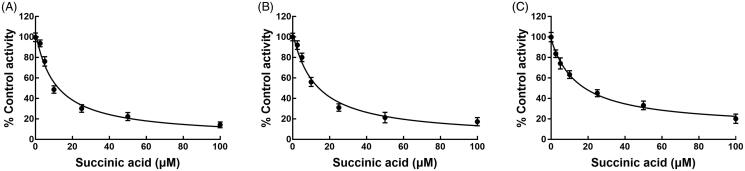
Dose-dependent experiment of CYP 3A4 (A), 2D6 (B), and 2C9 (C) in the presence of 0, 2.5, 5, 10, 50, and 100 μM succinic acid.

The activity of CYP3A4, 2D6, and 2C9 was significantly inhibited after incubating with succinic acid (*p* < 0.05, Figure 2). The administration of 100 μM succinic acid inhibited the activity of CYP3A4 to 14.12%, and the value of IC_50_ was obtained as 12.82 μM (Figure 3(A)). Similarly, the inhibition of CYP2D6 and 2C9 was also performed in a concentration-dependent manner with the IC_50_ values of 14.53 and 19.60 μM, respectively (Figure 3(B, C)).

**Figure 4.**
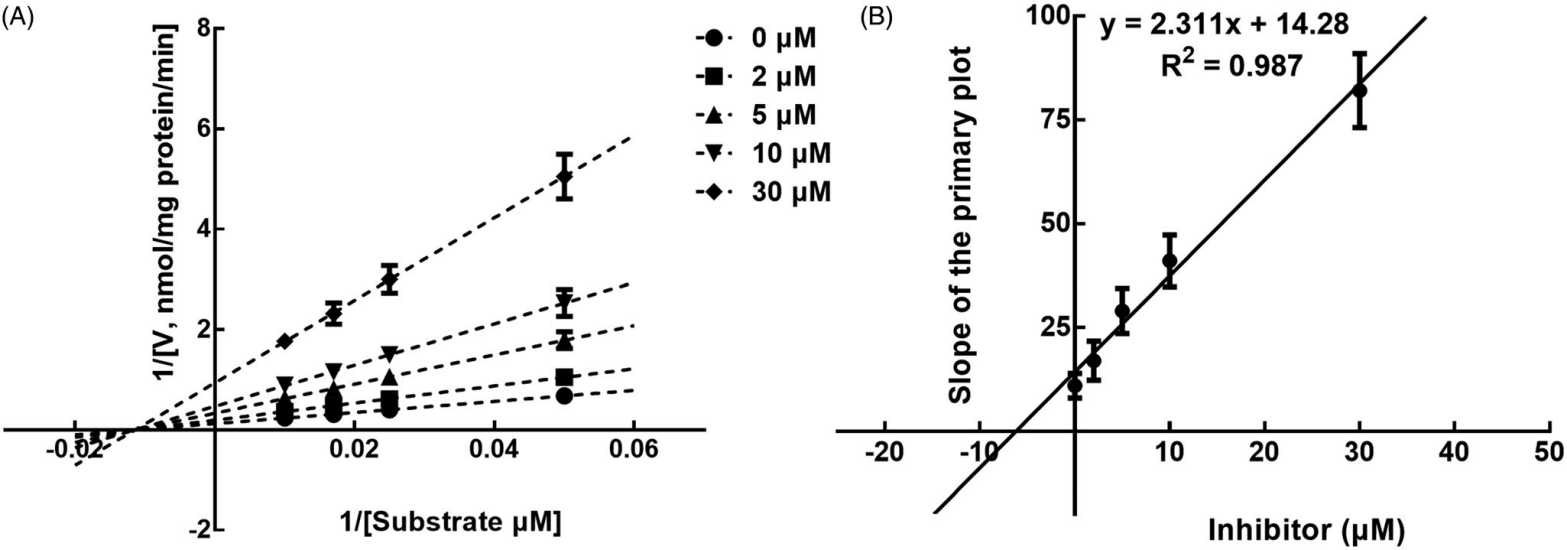
Lineweaver-Burk plots (A) and the secondary plot for Ki (B) of inhibition of succinic acid on CYP3A4 catalysed reactions (testosterone 6β-hydroxylation) in pooled HLM. Data are obtained from a 30 min incubation with testosterone (20–100 μM) in the absence or presence of succinic acid (0–30 μM). All data represent the mean of the incubations (performed in triplicate).

**Figure 5.**
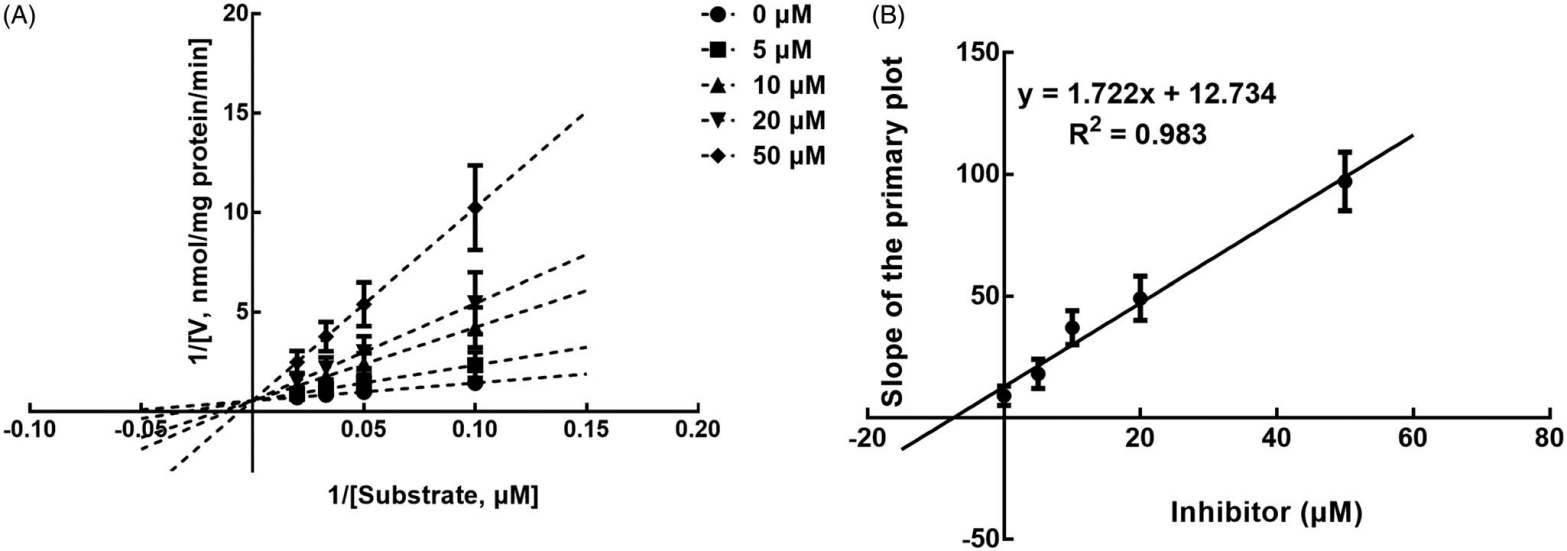
Lineweaver-Burk plots (A) and the secondary plot for Ki (B) of inhibition of succinic acid on CYP2D6 catalysed reactions (dextromethorphan *O*-demethylation) in pooled HLM. Data are obtained from a 30 min incubation with dextromethorphan (10–50 μM) in the absence or presence of succinic acid (0–50 μM). All data represent the mean of the incubations (performed in triplicate).

**Figure 6.**
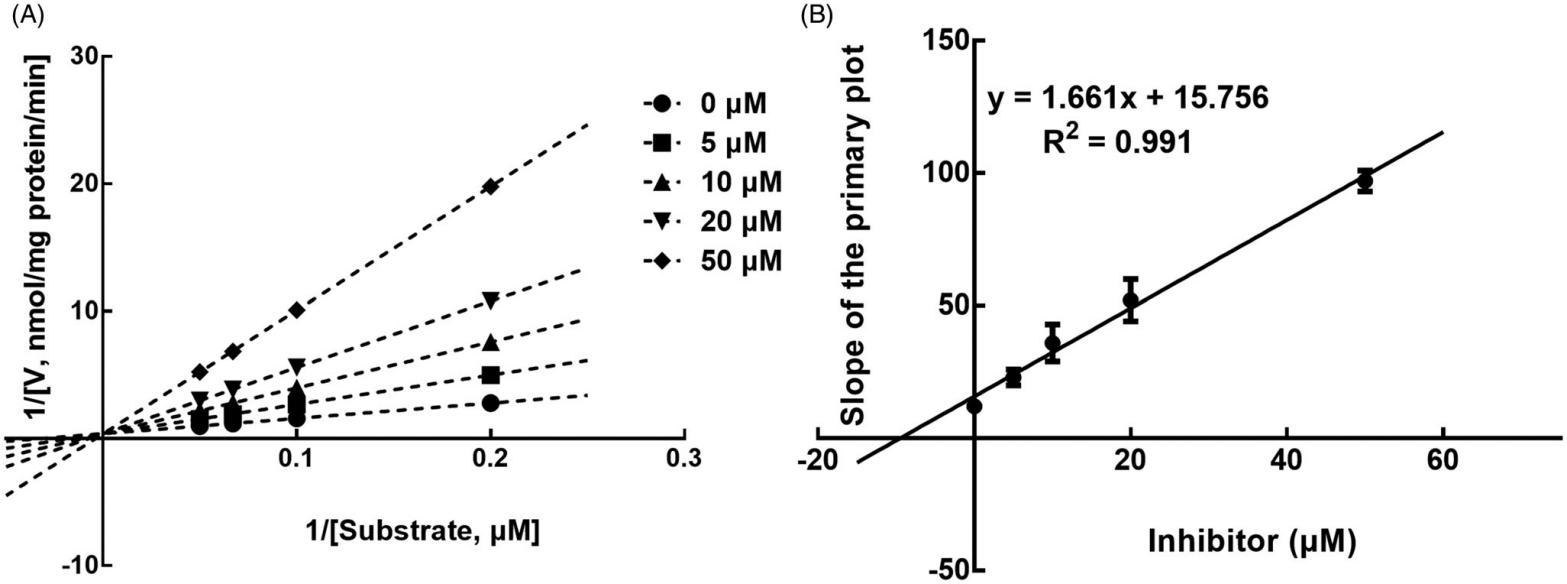
Lineweaver-Burk plots (A) and the secondary plot for Ki (B) of inhibition of succinic acid on CYP2C9 catalysed reactions (diclofenac 4'-hydroxylation) in pooled HLM. Data are obtained from a 30 min incubation with diclofenac (5–20 μM) in the absence or presence of succinic acid (0–50 μM). All data represent the mean of the incubations (performed in triplicate).

The results from the Lineweaver-Burk plots showed that the inhibition of CYP3A4 was best fitting with the non-competitive model with the *Ki* value of 6.18 μM (Figure 4). The inhibition of CYP2D6 and 2C9 was shown to be conducted in a competitive manner with the *Ki* value of 7.40 and 9.48 μM, respectively (Figures 5 and 6).

### The inhibition of CYP3A4 was time-dependent

**Figure 7.**
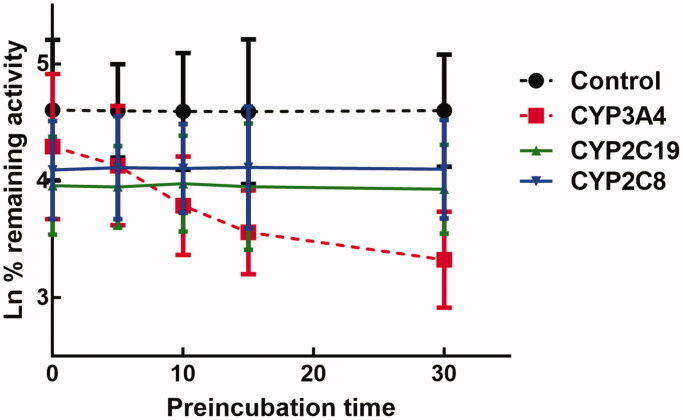
Effect of incubation time on the inhibition of CYP3A4, 2D6, and 2C9.

**Figure 8.**
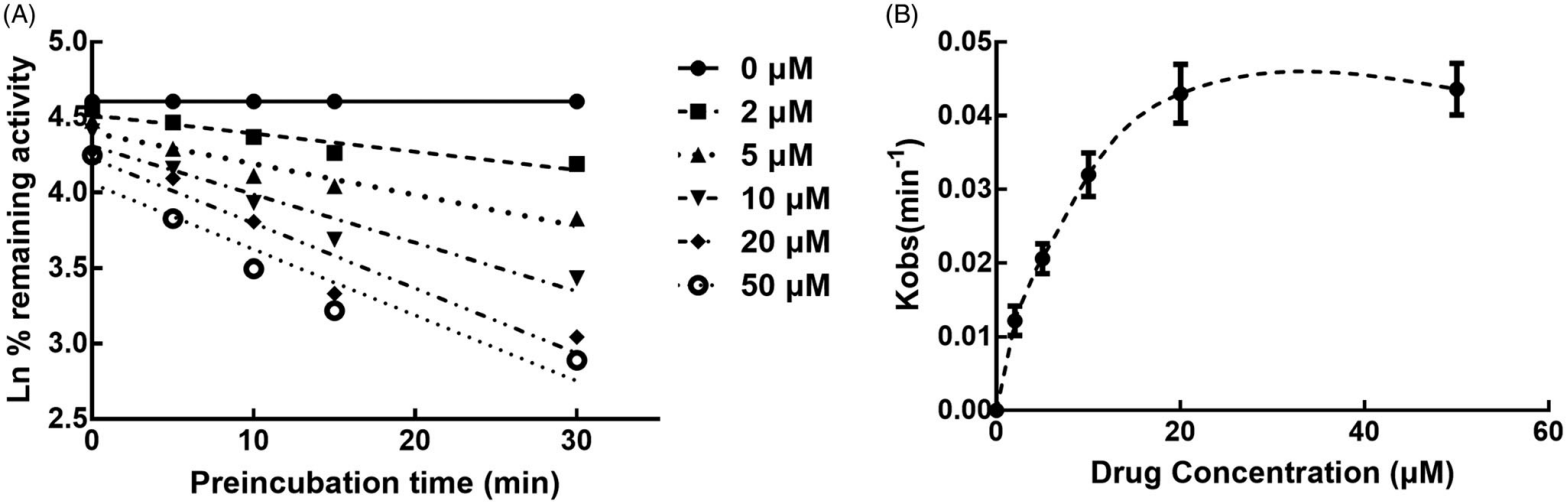
Time and concentration-inactivation of microsomal CYP3A4 activity by succinic acid in the presence of NADPH. The initial rate constant of inactivation of CYP3A4 by each concentration (K_obs_) was determined through linear regression analysis of the natural logarithm of the percentage of remaining activity versus pre-incubation time (A). The *KI* and *K_inact_* values were determined through non-linear analysis of the Kobs versus the pachymic acid concentration (B).

The activity of CYP3A4 was decreased to 26.65% after incubating with succinic acid for 30 min, which indicated that inhibition of CYP3A4 by succinic acid was performed in a time-dependent manner. While the activity of CYP2D6 and 2C9 was not affected by the incubation time (Figure 7). For the time-dependent inhibition of CYP3A4, the corresponding parameters *KI*/K*_inact_* was 6.52/0.051 min/μM, which suggested that about 5.1% CYP3A4 came inactivated by succinic acid (Figure 8).

## Discussion

In traditional Chinese medicine, it is common to combine two or more types of drugs in the clinic, which can make the treatment more efficient. The effects of drugs on the activity of CYP450 have drawn special attention in previous studies, which provides reference for the potential interaction between different drugs. Therefore, drug-drug interaction is one of the most important factors which is associated with the pharmacokinetics of drugs. Succinic acid is a major extraction of amber, which is widely used in cardiovascular disease therapy. It is easier for succinic acid to be co-administrated with other drugs, which makes the drug-drug interaction possible.

The *in vitro* effect of succinic acid on the activity of CYPs was investigated in this study in the pooled HLMs. Succinic acid inhibited the activity of CYP3A4, 2D6, and 2C9 in a concentration-dependent manner. Succinic acid acted as a non-competitive inhibitor of CYP3A4 and a competitive inhibitor of CYP2D6 and 2C9, which might be caused by the similar structure of succinic acid and the substrates of CYP2D6 and 2C9. Moreover, the inhibition of CYP3A4 was affected by the incubation time. These results indicated in the potential drug-drug interaction between succinic acid and drugs metabolized by CYP3A4, 2D6, and 2C9, and the administration dosage and the incubation time are also two vital factors that affected the drug-drug interaction.

CYP1, CYP2, and CYP3 are three kinds of major CYP450s responsible for the metabolism of most drugs (Xu et al. 2018). CYP3A4 is one of the most important enzymes in the CYP3A family, which is involved in the metabolism of 50% drugs (Basheer and Kerem 2015; Srinivas 2016). The inhibition of CYP3A4 could induce the toxicity or failure of drugs. The inhibition of CYP3A4 by succinic acid indicated that succinic acid should be used carefully when combining with drugs metabolized by CYP3A4, and the intrinsic clearance rate may be affected by this kind of inhibition, which can predicate the clearance of drugs in the clinic (Chao et al. 2010). Although CYP2D6 and 2C9 accounts a little of CYP450 expressed in the liver, they still participate in the metabolism of various drugs, and CYP2D6 was known to be responsible for the genetic polymorphism (de Groot et al. 2009). The inhibition of CYP2D6 and 2C9 for the poor metabolizer population may be harmful since its inhibition may significantly increase drug concentration in the body, leading to severe adverse effects (Lymperopoulos et al. 2015).

The limitation of this study is that *in vivo* experiments are lacked, which is beneficial for the extensive understanding of the clinical application of succinic acid and the co-administration of succinic acid with other drugs. On the other hand, the effect of succinic acid on the other enzymes or proteins that play roles in the metabolism, transformation, or transport of drugs, such as *P-gp* and UDP-glucuronosyltransferases (UGT) (Romand et al. 2017; Mano et al. 2018; Yang et al. 2018; Vrba et al. 2020). In preclinical studies to evaluate pharmacokinetics and toxicity, animal models have been commonly used to replace humans (Shi et al. 2016). Therefore, further studies should pay more attention to the *in vivo* interactions and effects on the activity of *P-gp* and UGT.

## Conclusions

The *in vitro* inhibitory effect of succinic acid on the activity of CYP3A4, 2D6, and 2C9 was found in this research. The concentration of drugs and the incubation time are two important factors that influenced the activity of CYPs. These results indicated the potential interaction between succinic acid and drugs metabolized by CYP3A4, 2D6, and 2C9, but further *in vivo* studies are needed to verify these potential drug-drug interactions.
